# Primary healthcare delivery adaptations in war-induced population displacement

**DOI:** 10.1186/s13584-025-00698-0

**Published:** 2025-06-10

**Authors:** Assi Cicurel, Yael Wolff Sagy, Ilan Feldhamer, Shlomit Yaron, Shani Caspi-Regev, Doron Netzer, Ronen Arbel, Gil Lavie

**Affiliations:** 1 Southern district , Clalit Health Services, Beer‑Sheva, Israel; 2 Faculty of Health Sciences, Ben-Gurion University of the Negev, Beer‑Sheva, Israel; 3 Branch of Planning and Strategy, Clalit Health Services, Tel Aviv, Israel; 5 Schneider children’s medical center of Israel, Petah-Tikva, Israel; 6 Adelson School of Medicine, Ariel University, Ariel, Israel; 7 Community Medical Services Division, Clalit Health Services, Tel Aviv, Israel; 8 Maximizing Health Outcomes Research Lab, Sapir College, Sderot, Israel; 9 Ruth and Bruce Rappaport Faculty of Medicine, Technion - Israel Institute of Technology, Haifa, Israel

## Abstract

**Background:**

Impact of war on civilians in high-income countries has been relatively underexplored in research. Internal displacement of populations within a country during war challenges healthcare universal access, utilization, and continuity of care. Healthcare systems can prepare and adjust to mitigate detrimental effects. Therefore, our objective was to examine primary healthcare delivery adaptations during war-induced population displacement and the effects on primary healthcare utilization.

**Methods:**

Observational, repeated cross-sectional study based on Clalit Health Services (CHS) electronic medical records (EMR) data. Outcomes were the rates of visits in primary care during five months following the war, compared to the previous year, by population group. All CHS members were included, 4.86 million, classified into four groups: (1) evacuated municipalities in the South (ES); (2) evacuated municipalities in the North (EN); (3) areas of restricted activity (RA) (4) rest of the Country (RC). The considered exposures were the state of war and internal displacement of populations, extended periods of restricted activities for areas under threat, and primary healthcare delivery adaptation measures. The main outcomes and measures were primary care visit rates grouped into four consecutive weeks clusters. Visits were further classified as in-person or telehealth visits.

**Results:**

Healthcare delivery adaptation measures included fast set-up of pop-up primary clinics in evacuated population concentrations, services expansion (online visits 24/7, medication delivery range), and expanded services for internally displaced persons (designated call center lines and text-based nursing service). During the initial weeks following the outbreak of war overall visits declined, mainly in displaced populations (by 43.9% (95% CI: 42.2-45.6%) and 19.1% (95% CI: 17.1 − 21.1%) in the first month in ES and EN, respectively). Visits rates gradually recovered in all population groups, returning to baseline within 12 weeks. This was driven by a sharp initial decline of in-person visits, and attenuated by increased usage of telehealth, mainly observed in displaced populations.

**Conclusions:**

The outbreak of war and population displacement was associated with decreased primary care visits, while telehealth service utilization increased significantly. This increase was partly facilitated by telehealth consultations provided by patients’ regular primary care physicians, often themselves displaced, thereby preserving continuity of care through existing trust and rapport. Healthcare systems should proactively integrate telehealth solutions into emergency preparedness plans, prioritizing continuity of patient-provider relationships even during displacement. Future research is needed to evaluate the quality and equity implications of telehealth adaptations and their impact on long-term health outcomes.

**Supplementary Information:**

The online version contains supplementary material available at 10.1186/s13584-025-00698-0.

## Background

### Introduction

The impact of conflicts on health and healthcare utilization is a subject of increasing relevance and concern globally. Research worldwide consistently showed that war adversely affects health outcomes for diverse populations, beyond the direct detrimental effects of violence [1–8]. A specific group with unique circumstances during war is internally displaced persons. Challenges are often pronounced due to their vulnerable status and limited access to healthcare services. Studies indicate that internally displaced persons frequently endure poorer health outcomes compared to the general affected population due to reduced healthcare access and the influence of social determinants on their health needs. Consequently, the challenges faced by persons who were displaced from their homes in conflict zones require specialized attention, in order to ensure that their unique health needs are adequately and effectively addressed [9–12]. 

Service adaptations for addressing healthcare needs of internally displaced persons in conflict zones described in the literature include the use of remote/digital health services, mobile clinics, health facility-based and community-based care, as well as outreach and home visits [13–19]. The large-scale invasion of Ukraine by Russia in February 2022 triggered an unprecedented displacement crisis, and the sudden migration placed significant strain on primary healthcare systems in host nations. Refugees faced multiple healthcare challenges, including disrupted continuity of care, barriers to accessing primary healthcare services, increased risks of communicable disease outbreaks, and high rates of mental health disorders. A major concern in the refugee response has been continuity of chronic disease management, as disrupted healthcare services risk worsening pre-existing conditions [20]– [21].

Studies and reviews on healthcare services and the consequences of war on internally displaced persons predominantly focus on low- and middle-income countries. In Gaza, population displacement is one healthcare challenge that adds to other immediate detrimental effects of war. These are faced by a local healthcare system suffering from inherent structural weaknesses, including centralized care-delivery by hospitals, insufficient preventive and primary care medicine, as well as dependency on outside assistance [22]. Evidence concerning war conflicts and healthcare system response in high-income countries with advanced healthcare systems is relatively limited. This highlights the need for more comprehensive research in high-income countries to better understand and address the healthcare challenges faced by internally displaced persons in these environments.

### CHS community health services response to war

Israel is at war since 07/10/2023. Following an attack by Hamas organization that included high trajectory indirect fire, direct assaults, killing, and kidnapping of civilians in the south, and indirect fire in northern Israel, persons residing in the most affected areas were evacuated to hotels or hostels in less-threatened areas of the country within the first few weeks of the war. War effected all parts of the country, but a gradient of disruption of normal life was evident with four distinct groups of effect (see Table 1).

**Table 1:** Healthcare system adaptation measures

	CHS members from evacuated municipalities in the South, *n* = 40,362	CHS members from evacuated municipalities in the North, *n* = 51,023	CHS members in areas of “restricted activity”, *n* = 789,842	CHS members in the rest of the country, *n* = 3,983,985
Description	Direct invasion and intense high trajectory fire	Highly intense high trajectory fire	Intense high trajectory fire	Lower intensity of high trajectory fire
Effect	Emergency evacuation	Planned evacuation	Stay at home disruption of activities	Return to normal activity
**System adaptation measures**				
Fast set-up of pop-up primary clinics	Yes	Yes	No	No
Call center*- designated lines for internally displaced patients	Yes	Yes	No	No
Call center- text-based nursing service for internally displaced patients	Yes	Yes	No	No
Facilitated guest service in all clinics	Yes	Yes	Partial	Partial
Expanded access** to patients’ EMR	Yes	Yes	Yes	Yes
Expanded medication delivery services	Yes	Yes	Yes	Yes
Expanded hours of online visits to 24/7	Yes	Yes	Yes	Yes

*Nursing and administrative service

**Access of primary care physicians to patients EMRs was granted regardless of patients’ address and clinic, to facilitate continuity of care

Patients from areas bordering hostilities in southern Israel that suffered from high trajectory and direct invasion to communities with casualties were urgently evacuated, sometimes under fire, with no time to prepare. Many patients left their homes with no medications, documents, or necessary medical equipment (evacuated south ES). Patients from areas bordering hostilities in northern Israel that included only indirect high trajectory fire were evacuated within days in an organized manner which enabled preparation and adaptation of primary care services to evacuation of patients (evacuated north EN). Patients from areas further away from hostilities but who suffered intense high trajectory fire were not evacuated. However, the civilian population was instructed to remain at home and in proximity to shelters and schools were closed, hence leading to a major disruption of normal life for several weeks (restricted activity RA). Other areas of the country suffered less disruption of daily life, despite high trajectory fire, with normal activities returning within days (rest of the country, RC).

One day following the attack, primary care services were provided in all areas that were deemed as safe for work and in clinics with functional bomb shelters within the clinic. In order to facilitate access to in-person services for internally displaced populations, emergency popup primary care clinics were established in or around the hotels that received evacuated patients, offering walk-in care with physicians, nurses, administrative staff, and pharmacy services. Additionally, complete access to guest walk-in or same day in-person primary care appointments in any clinic around the country was facilitated. To allow efficient EMR-based service, computerized health records that are normally open only to the personal primary care team or with personal card swipe were opened for access to all primary care physicians.

Telehealth services were well established prior to the outbreak of Oct 7, 2023 war. However, some of these services were expanded to address the needs of those displaced, including Call center designated lines providing both administrative services and nurse consultations, and unique text-based nurse consultations. To promote continuity of care, whenever possible, internally displaced patients remained under the responsibility of their regular primary care physician. Other pre-existing services were expanded to the advantage of all CHS members, including the geographical expansion of medication delivery services, and 24 h per day/ 7 days per week availability of online consultations with primary care physicians.

### Objective

This study examined primary healthcare delivery adaptations during war-induced population displacement and their effects on primary healthcare utilization, including in-person and telehealth encounters over a period of five months, by circles of influence.

## Methods

### Study design

This observational, repeated cross-sectional population-based study was based on data obtained from the electronic medical records of Clalit healthcare services (CHS). In Israel, health insurance is universal and mandatory, and all residents are required to choose one of four health maintenance organizations. CHS is the largest healthcare organization in Israel, which insures 4.87 million patients (52% of the population). In the areas where population-displacement occurred, CHS insures nearly 70% of the population. Primary healthcare services, including in-person and telehealth visits (mostly conducted as phone calls) are freely available to all members.

The study outcomes were the rates of visits in primary care, further classified as in-person or telehealth visits. Data on visits rates were extracted from October, 16 2022 until February 28, 2024. The starting date for the study was chosen according to the Jewish calendar as the start of the first full working week after the holiday of Sukkot (October 16, 2022), to allow a parallel comparison with regard to national holidays versus the period starting on October 8, 2023. The exposure was the state of war and internal displacement of populations, extended periods of restricted activities for areas under threat, and primary healthcare delivery adaptation measures were taken by CHS.

### Study population

All CHS members were included, and classified into four groups of expected circles of influence, defined by their address of permanent residence: (1) evacuated municipalities in the South (ES); (2) evacuated municipalities in the North (EN); (3) areas of that were confined to restrictions to daily activities, including the closure of schools as defined by the Israeli Home Front Command during October 2023 (RA); (4) rest of the country (RC).

### Statistical analysis

Visit rates were grouped into clusters of four-consecutive weeks, and parallel calendar periods before and after October 7th, 2023 were compared. Calendar periods were defined according to the Hebrew-calendar, to account for the influence of holidays on healthcare services availability. To assess the differences in the effect of exposure between study groups, incidence rate ratios (IRR) with their 95% confidence intervals (CI) were calculated for the difference in visits rates before and after October 7, 2023, using Poisson regression models. Analyses were also performed stratified by three age groups: 0–17 years old, 18–64 years old, and 65 years or older.

## Results

### Figures legend

Visits rates during the previous period (October 2022- February 2023) are represented by black dots; visits rates during the current period (October 2023- February 2024) are represented by red dots; all visit rates are enumerated using the left axis (visits rate per 100 members). Percent change between periods are represented as blue bars with their 95% CIs and enumerated using the right axis (percent change).

### Study population

The study population included all 4.865 Million CHS members, 50.7% of them female.

The median age of the entire population was 30 years (IQR: 24–36 years) The proportion of patients aged 65 years or older was somewhat lower among patients from evacuated municipalities in the South (12.5%) and from areas of restricted activities (12.1%) compared to the rest of the country (13.7%).

Evacuated municipalities both in the South and in the North were characterized by a small proportion of low-SES areas of residence compared to the rest of the country (19% in the South and 15.9% in the North, compared to 39% in the rest of the country). Additionally, evacuated municipalities in the South had a higher proportion of high-SES areas of residence (43.3% classified as high SES) compared to the rest of the country (33.3% classified as high SES).

Patients from evacuated municipalities in the South had lower rates of comorbidities compared to patients from other areas for all comorbidities assessed, except for asthma which more frequent in this group (9.4% compared to 7.8% in the rest of the country). (Table 2)

**Table 2:** Population characteristics

Patients Characteristics		evacuated municipalities in the South, *n* = 40,362	evacuated municipalities in the North, *n* = 51,023	areas of “restricted activity”, *n* = 789,842	rest of the country, *n* = 3,983,985	Total, *n* = 4,865,212
		*n* (%)	*n* (%)	*n* (%)	*n* (%)	*n* (%)
Sex, female		20,185 (50%)	25,771 (50.5%)	402,219 (50.9%)	2,020,045 (50.7%)	2,468,220 (50.7%)
Age, median (IQR)		30 (25, 35)	33 (26, 40)	28 (23, 33)	30 (24, 36)	30 (24, 36)
Age group, years	0–17	14,405 (35.7%)	15,413 (30.2%)	294,098 (37.2%)	1,359,336 (34.1%)	1,683,252 (34.6%)
	18–44	13,859 (34.3%)	17,754 (34.8%)	268,162 (34%)	1,373,909 (34.5%)	1,673,684 (34.4%)
	45–64	7064 (17.5%)	10,830 (21.2%)	131,759 (16.7%)	703,630 (17.7%)	853,283 (17.5%)
	65+	5034 (12.5%)	7026 (13.8%)	95,823 (12.1%)	547,110 (13.7%)	654,993 (13.5%)
Socioeconomic status index	low (1–4)	7413 (19%)	7965 (15.9%)	288,331 (39.4%)	1,460,320 (38.5%)	1,764,029 (38.3%)
	medium (5–6)	14,771 (37.8%)	27,705 (55.2%)	249,440 (34.1%)	1,057,143 (27.9%)	1,349,059 (29.3%)
	high (7–10)	16,923 (43.3%)	14,478 (28.8%)	192,382 (26.3%)	1,266,605 (33.4%)	1,490,388 (32.3%)
	missing	1 (0%)	37 (0.1%)	1609 (0.2%)	5853 (0.2%)	7500 (0.2%)
Smoking status	current smoker	4594 (14.1%)	7952 (18.4%)	92,975 (14.8%)	447,998 (13.8%)	553,519 (14%)
	past smoker	4766 (14.6%)	6470 (15%)	69,716 (11.1%)	383,344 (11.8%)	464,296 (11.8%)
	never	23,274 (71.3%)	28,851 (66.7%)	463,782 (74%)	2,409,096 (74.3%)	2,925,003 (74.2%)
Charlson co-morbidity index	0	25,173 (68.5%)	33,639 (69.7%)	509,521 (69.6%)	2,651,399 (71.3%)	3,219,732 (71%)
	1–2	8807 (24%)	10,862 (22.5%)	165,602 (22.6%)	785,013 (21.1%)	970,284 (21.4%)
	3–4	1792 (4.9%)	2452 (5.1%)	35,985 (4.9%)	182,594 (4.9%)	222,823 (4.9%)
	5+	977 (2.7%)	1318 (2.7%)	20,770 (2.8%)	102,110 (2.7%)	125,175 (2.8%)
Obesity (BMI ≥ Kg/m2)		7128 (27.9%)	11,213 (33.4%)	161,950 (33%)	756,423 (30.2%)	936,714 (30.7%)
Diabetes		3020 (11.8%)	4389 (13.1%)	62,707 (12.8%)	338,113 (13.5%)	408,229 (13.4%)
Ischemic heart disease		1552 (6.1%)	2343 (7%)	30,709 (6.3%)	175,888 (7%)	210,492 (6.9%)
Congestive heart failure		441 (1.7%)	722 (2.1%)	10,332 (2.1%)	52,976 (2.1%)	64,471 (2.1%)
Asthma		2403 (9.4%)	2257 (6.7%)	36,152 (7.4%)	195,524 (7.8%)	236,336 (7.7%)
Chronic obstructive pulmonary disease		446 (1.7%)	794 (2.4%)	10,095 (2.1%)	56,332 (2.2%)	67,667 (2.2%)
Malignancy		112 (0.4%)	173 (0.5%)	2073 (0.4%)	13,205 (0.5%)	15,563 (0.5%)

### Overall visits in primary care

**Fig. 1:**
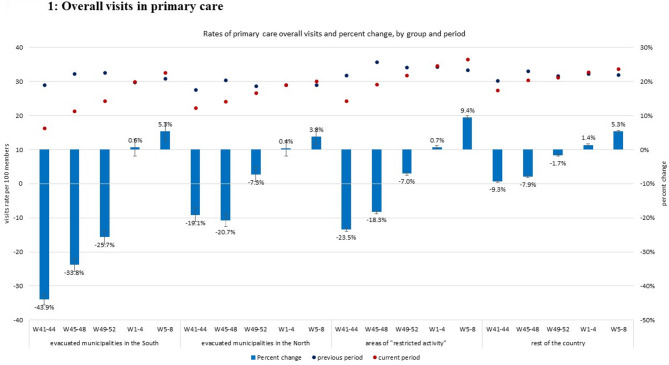
Overall visits in primary care. Black and red dots represent 4-weeks visits rates per 100 members during war (2023, red) and during the baseline comparison period (2022, black); blue bars represent the percent change between periods, with 95% confidence intervals represented by error bars

The rates of overall visits in primary care sharply decreased in the first month after October 7, in all study groups. This decrease gradually attenuated in the next months, with overall visits rates returning to baseline in the fourth month of follow-up. The decrease was the sharpest in patients from evacuated municipalities in the South, lower by 43.9% (95% CI: 42.2-45.6%) compared to baseline in the first month, by 33.8% (95% CI: 32.0- 35.6%) in the second month, and by 25.7% (95% CI: 23.7-27.6%) in the third month. In the fourth month, visits rates were similar to baseline, and in the fifth month they were higher by 5.3% (95% CI: 2.8-8.0%) compared to baseline. In patients from evacuated municipalities in the North, the rates of overall visits in primary care were lower than baseline in the first and second month by 19.1% (95% CI: 17.1 − 21.1%) and 20.7% (95% CI: 18.8-22.6%), respectively. This decline attenuated in the third month, with 7.3% (95% CI: 5.1-9.4%) less visits than in baseline, and ceased by the fourth month, when visits rates were similar to baseline. In the fifth month, visits rates were slightly higher than baseline, by 3.8% (95% CI: 1.5-6.2%) (Fig. 1).

In patients residing in areas of “restricted activity”, a sharp decline in the rates of overall visits was also observed in the first month, by 23.5% (23.0-23.9%), although less than the decline observed in residents of the evacuated areas. This decrease gradually attenuated in the following months, and by the fourth month, visits rates in this group were similar to those observed in the previous year. In patients residing in the rest of the country, the observed decline in in-person primary visits rates in the first month was 9.3% (9.0-9.5%) compared to the previous year. This decline gradually attenuated in the following months and a return to baseline rates was observed by the fourth month (Fig. 1).

### In-person visits in primary care

**Fig. 2:**
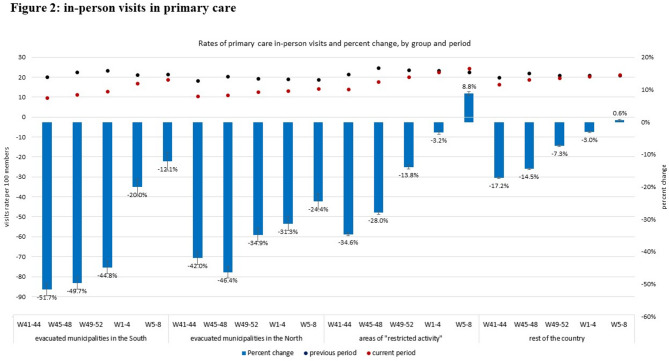
In-person visits in primary care. Black and red dots represent 4-weeks visits rates per 100 members during war (2023, red) and during the baseline comparison period (2022, black); blue bars represent the percent change between periods, with 95% confidence intervals represented by error bars

In patients from evacuated municipalities in the South, the rates of in-person visits in primary care sharply decreased after October 7th by 51.7% (95% CI: 49.8-53.5%) in the first month. This decrease attenuated thereafter but remained lower than in the previous (comparison) period by 49.7% (47.8-51.4%), 44.8% (42.9-46.7%), 20.0% (17.4-22.5%), and 12.1% (9.3- 14.7%), in the second, third, fourth, and fifth following months, respectively. In patients from evacuated municipalities in the North, the rates of in-person visits in primary care have also decreased sharply in the first month, by 42.0% (40.0-43.9%), but the sharpest decrease in visits was observed in the second month, with 46.4% (44.6-48.1%) less visits than in the parallel period in the previous year. During the following months this decrease gradually attenuated. However, by the fifth month, the rates of in-person visits were still lower compared to those in the previous year by 24.4% (22.1-26.7%) (Fig. 2).

In patients residing in areas of “restricted activity”, a sharp decline in the rates of in-person visits was also observed in the first month, by 34.6% (34.1-35.1%), although less than the decline observed in residents of the evacuated areas. This decrease gradually attenuated in the following months. By the Fifth month, visits rates in this group exceeded those observed in the previous year by 8.8% (8.1-9.5%). In patients residing in the rest of the country, a decline in in-person primary visits rates of 17.2% (17.1-17.3%) in the first month compared to the previous year. This decline gradually attenuated in the following months and a return to baseline rates was observed by the fifth month (Fig. 2).

### Telehealth visits in primary care

In patients from evacuated municipalities in the South, the rates of telehealth visits in primary care also decreased in the first month after October 7th (by 26.4%). However, starting from the second month and onwards, telehealth visits steadily increased compared to the parallel period in the previous year. This increase reached a peak of 50.9% more telehealth visits in the fourth month. In patients from evacuated municipalities in the North, the use of telehealth primary care visits sharply increased after October 7th compared to the previous year, starting from the first month with 25.4% increase, gradually reaching a 62.2% increase in telehealth visits compared to the previous year in the fourth month. This increased used of telehealth somewhat attenuated in the fifth month.

**Fig. 3:**
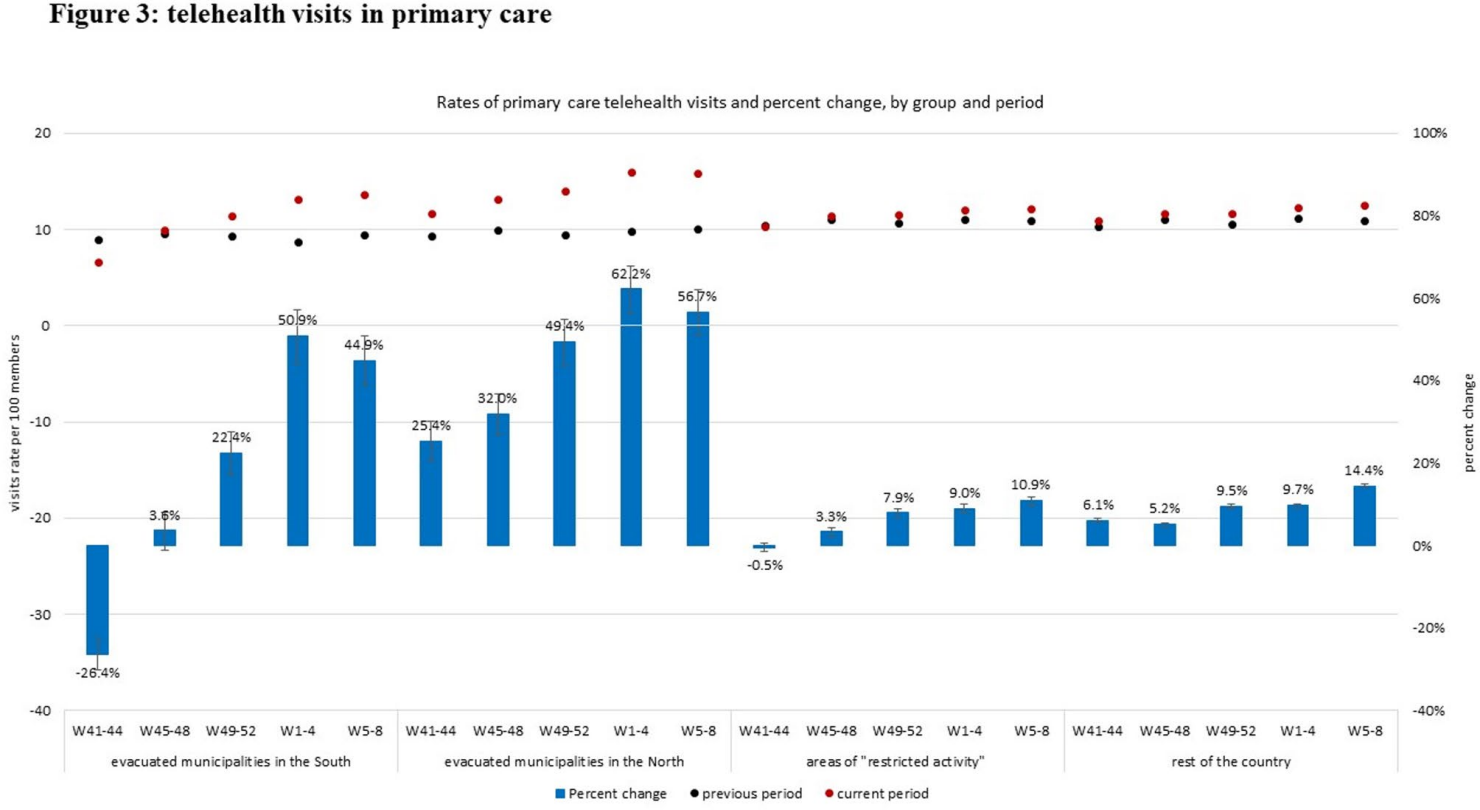
Telehealth visits in primary care. Black and red dots represent 4-weeks visits rates per 100 members during war (2023, red) and during the baseline comparison period (2022, black); blue bars represent the percent change between periods, with 95% confidence intervals represented by error bars

In areas of restricted activity and in the rest of the country, the use of primary care telehealth visits also increased compared to the previous year. However, this increase was moderate (up to 14% compared to the previous year) compared to that observed in patients from evacuated municipalities (Fig. 3).

### Stratification by age-group

Stratification by age group (0–17 years old, 18–64 years old, and 65 years or older) revealed that the sharpest decreases in overall visits across all areas were in children; with decreases ranging from − 11% in the rest of the country and up to -43% in evacuated municipalities in the South (mean change during the study period), while in adults the mean changes were subtle (Table S1).

Moreover, while telehealth services use increased in most areas from the first week of the war for adults, in children, increased use of telehealth was observed only in the third month of the war in the different areas (Table S1).

## Discussion

In this observational study involving nearly five million individuals, we documented significant disruptions in primary healthcare utilization immediately following the onset of conflict-induced internal displacement. Overall visits in primary care dropped as war erupted, driven by a sharp decline of in-person visits, and mitigated by an increased usage of telehealth services, mainly in displaced populations. Primary care utilization showed a gradient of disruption that corresponded with the severity of disturbance to normal life.

Consistent with global literature on conflict-related healthcare disruptions, our findings highlight a sharp initial reduction in primary care visits predominantly driven by reduced in-person consultations, aligning with studies demonstrating disrupted healthcare access among internally displaced populations in various conflict zones globally [9, 12].

The observed gradient of disruption across population groups underscores the importance of tailored health service adaptations responsive to the severity of life disruptions experienced by affected populations. While general populations resumed baseline healthcare utilization within months, displaced groups experienced prolonged reductions in in-person care utilization. This discrepancy emphasizes the vulnerability and unique healthcare needs of internally displaced persons (IDPs), corroborating existing evidence that highlights sustained healthcare access barriers faced by IDPs, even within high-income settings [23]– [24].

Telehealth services emerged as a critical adaptive strategy in response to displacement-related barriers. The marked increase in telehealth use among displaced populations underscores its potential to maintain continuity of care under emergency conditions, an observation supported by prior evidence from both COVID-19 [25–27] and humanitarian crises [28]– [29]. Importantly, some of this increased telehealth utilization was facilitated by consultations conducted by patients’ regular primary care physicians, who continued providing care remotely, thereby leveraging established trust and pre-existing patient-physician rapport. In several cases, both patients and physicians were internally displaced yet maintained their therapeutic relationships remotely, emphasizing the critical importance of preserving continuity of care even during displacement.

However, the increased reliance on telehealth prompts critical questions about care quality, equity, and long-term sustainability. While telehealth facilitated immediate care access, particularly chronic disease management, concerns persist regarding its effectiveness in comprehensive clinical assessments and in meeting complex healthcare needs of IDPs. Future research should explore long-term health outcomes associated with telehealth utilization during displacement, specifically investigating quality of care, patient satisfaction, and equity of access.

## Conclusions

In conclusion, healthcare systems in high-income countries must integrate flexible telehealth strategies within emergency preparedness frameworks. Maintaining continuity of care, especially through telehealth provided by familiar healthcare providers should be prioritized to leverage established trust and relationship. Addressing structural and operational challenges of telehealth—such as digital literacy, connectivity, and equitable access—will be essential. Future policy and research initiatives that focus on optimizing telehealth interventions are likely to enhance resilience in healthcare systems confronting ongoing or future displacement crises.

## Electronic supplementary material

Below is the link to the electronic supplementary material.

Supplementary Material 1

## Data Availability

Due to Clalit Health Services data privacy regulations and as per the institutional Helsinki and data utilization committee approvals for this study, the patient-level data used for this study cannot be shared.
