# Supplementary material for: Primary healthcare delivery adaptations in war-induced population displacement
Source: Isr J Health Policy Res. 2025 Jun 10;14:35. doi: 10.1186/s13584-025-00698-0 (PMC12150444; doi:10.1186/s13584-025-00698-0)
Supplement: Supplementary file 1 — Supplementary Material 1 [file 13584_2025_698_MOESM1_ESM.docx]

## Supplementary tables

## Visual summary:

## S1: Change in primary care visit rates (overall, in-person, and telehealth), by age groups

| Age group / population group | Week | **Rates of overall visits in primary care** | | **Percent change (95% CI)** |
| --- | --- | --- | --- | --- |
|  |  | **previous period** | **current period** |  |
| **Ages 0-17 years** | | | | |
| evacuated municipalities in the South | W41-44 | 30.0 | 9.8 | -0.67 (-0.69--0.65) |
|  | W45-48 | 34.8 | 13.4 | -0.62 (-0.64--0.59) |
|  | W49-52 | 35.7 | 18.1 | -0.49 (-0.52--0.47) |
|  | W1-4 | 28.3 | 23.4 | -0.17 (-0.21--0.13) |
|  | W5-8 | 31.2 | 24.5 | -0.21 (-0.25--0.18) |
| evacuated municipalities in the North | W41-44 | 26.9 | 13.6 | -0.5 (-0.52--0.47) |
|  | W45-48 | 31.6 | 16.0 | -0.49 (-0.52--0.47) |
|  | W49-52 | 30.0 | 18.1 | -0.4 (-0.42--0.37) |
|  | W1-4 | 30.6 | 21.3 | -0.3 (-0.33--0.27) |
|  | W5-8 | 28.9 | 23.8 | -0.17 (-0.21--0.14) |
| areas of "restricted activity" | W41-44 | 28.2 | 14.9 | -0.47 (-0.48--0.47) |
|  | W45-48 | 34.2 | 20.6 | -0.4 (-0.4--0.39) |
|  | W49-52 | 32.0 | 25.1 | -0.21 (-0.22--0.21) |
|  | W1-4 | 31.8 | 30.4 | -0.04 (-0.05--0.03) |
|  | W5-8 | 28.8 | 33.5 | 0.16 (0.15-0.18) |
| rest of the country | W41-44 | 27.1 | 18.2 | -0.33 (-0.33--0.33) |
|  | W45-48 | 31.0 | 25.3 | -0.19 (-0.19--0.18) |
|  | W49-52 | 29.1 | 26.6 | -0.08 (-0.09--0.08) |
|  | W1-4 | 29.4 | 28.7 | -0.02 (-0.03--0.02) |
|  | W5-8 | 27.8 | 29.9 | 0.07 (0.07-0.08) |
| **Ages 18-64 years** | | | | |
| evacuated municipalities in the South | W41-44 | 24.3 | 16.3 | -0.33 (-0.36--0.3) |
|  | W45-48 | 26.6 | 21.3 | -0.2 (-0.23--0.17) |
|  | W49-52 | 27.1 | 23.1 | -0.15 (-0.18--0.12) |
|  | W1-4 | 26.0 | 29.0 | 0.11 (0.07-0.16) |
|  | W5-8 | 26.2 | 32.1 | 0.23 (0.18-0.27) |
| evacuated municipalities in the North | W41-44 | 23.0 | 22.3 | -0.03 (-0.07-0) |
|  | W45-48 | 25.7 | 22.8 | -0.12 (-0.14--0.09) |
|  | W49-52 | 24.2 | 25.0 | 0.03 (0-0.07) |
|  | W1-4 | 24.6 | 27.2 | 0.1 (0.07-0.14) |
|  | W5-8 | 25.0 | 27.5 | 0.1 (0.07-0.14) |
| areas of "restricted activity" | W41-44 | 28.5 | 25.3 | -0.11 (-0.12--0.11) |
|  | W45-48 | 31.2 | 28.8 | -0.08 (-0.08--0.07) |
|  | W49-52 | 30.4 | 30.2 | 0 (-0.01-0) |
|  | W1-4 | 30.8 | 31.6 | 0.03 (0.02-0.03) |
|  | W5-8 | 30.7 | 33.1 | 0.08 (0.07-0.09) |
| rest of the country | W41-44 | 25.9 | 26.6 | 0.02 (0.02-0.03) |
|  | W45-48 | 28.1 | 27.3 | -0.03 (-0.03--0.03) |
|  | W49-52 | 27.2 | 27.4 | 0.01 (0-0.01) |
|  | W1-4 | 28.0 | 28.8 | 0.03 (0.02-0.03) |
|  | W5-8 | 28.1 | 29.9 | 0.06 (0.06-0.07) |
| **Ages 65 years or older** | | | | |
| evacuated municipalities in the South | W41-44 | 45.5 | 33.6 | -0.26 (-0.31--0.21) |
|  | W45-48 | 48.1 | 42.2 | -0.12 (-0.17--0.07) |
|  | W49-52 | 47.0 | 45.1 | -0.04 (-0.09-0.02) |
|  | W1-4 | 48.6 | 51.3 | 0.06 (0-0.11) |
|  | W5-8 | 49.0 | 55.7 | 0.14 (0.08-0.2) |
| evacuated municipalities in the North | W41-44 | 45.8 | 39.4 | -0.14 (-0.18--0.1) |
|  | W45-48 | 46.5 | 45.7 | -0.02 (-0.06-0.03) |
|  | W49-52 | 43.4 | 49.6 | 0.14 (0.09-0.2) |
|  | W1-4 | 42.5 | 51.5 | 0.21 (0.16-0.27) |
|  | W5-8 | 44.6 | 52.4 | 0.18 (0.12-0.23) |
| areas of "restricted activity" | W41-44 | 54.4 | 46.9 | -0.14 (-0.15--0.13) |
|  | W45-48 | 57.8 | 54.8 | -0.05 (-0.06--0.04) |
|  | W49-52 | 55.6 | 56.7 | 0.02 (0.01-0.03) |
|  | W1-4 | 55.4 | 58.2 | 0.05 (0.04-0.06) |
|  | W5-8 | 56.6 | 58.5 | 0.03 (0.02-0.05) |
| rest of the country | W41-44 | 52.7 | 51.7 | -0.02 (-0.02--0.01) |
|  | W45-48 | 55.4 | 53.6 | -0.03 (-0.04--0.03) |
|  | W49-52 | 53.5 | 54.8 | 0.03 (0.02-0.03) |
|  | W1-4 | 53.7 | 55.9 | 0.04 (0.04-0.05) |
|  | W5-8 | 54.7 | 55.8 | 0.02 (0.01-0.02) |
